# Raloxifene augmentation in men and women with a schizophrenia spectrum disorder: A study protocol

**DOI:** 10.1016/j.conctc.2020.100681

**Published:** 2020-11-25

**Authors:** Bodyl A. Brand, Janna N. de Boer, Sebastianus B.J. Oude Ophuis, Margot I.E. Slot, Bieke De Wilde, Kirsten C.E.E.R. Catthoor, Angelique J. Goverde, P. Roberto Bakker, Machteld C. Marcelis, Koen P. Grootens, Jurjen J. Luykx, Sophie M. Heringa, Cynthia Shannon Weickert, Iris E.C. Sommer, Thomas W. Weickert

**Affiliations:** a Department of Psychiatry, UMC Utrecht Brain Center, University Medical Center Utrecht (UMCU), Utrecht University, Utrecht, the Netherlands; b Department of Biomedical Sciences and Systems, Cognitive Neurosciences, University of Groningen, University Medical Center Groningen (UMCG), Groningen, the Netherlands; c Ziekenhuis Netwerk Antwerpen (ZNA), Antwerp, Belgium; d The Collaborative Antwerp Psychiatric Research Institute (CAPRI), Antwerp, Belgium; e Department of Reproductive Medicine and Gynaecology, University Medical Center Utrecht (UMCU), Utrecht, the Netherlands; f Arkin, Institute for Mental Health, Amsterdam, the Netherlands; g Department of Psychiatry & Neuropsychology, School for Mental Health and Neuroscience, Maastricht University, Maastricht, the Netherlands; h Institute for Mental Health Care Eindhoven (GGzE), Eindhoven, the Netherlands; i Reinier van Arkel Institute for Mental Health Care (RvA), ‘s Hertogenbosch, the Netherlands; j Department of Translational Neuroscience, UMC Utrecht Brain Center, University Medical Center, Utrecht, Utrecht University, Utrecht, the Netherlands; k Outpatient Second Opinion Clinic, GGNet Mental Health, Warnsveld, the Netherlands; l Isala, Department of Medical Psychology, Zwolle, the Netherlands; m Neuroscience Research Australia, Randwick, New South Wales, Australia; n State University of New York, Upstate Medical University, Syracuse, NY, USA

**Keywords:** Antipsychotic medication, Estrogen, Raloxifene, Randomised controlled trial, Schizophrenia, AE, Adverse event, AF, alkaline phosphatase, ALAT, alanine aminotransferase, ANOVA, analysis of variance, ASAT, aspartate aminotransferase, APTT, activated partial thrombin time, BACS, Brief Assessment of Cognition in Schizophrenia, BDI, Beck's Depression Inventory, BNSS, Brief Negative Symptom scale, CRP, C-reactive protein, DHEA, dehydroepiandrosterone, DNA, Deoxyribonucleic acid, DSMB, Data Safety Monitoring Board, eGFR, estimated glomerular filtration rate, EQ-5D-5L, Euro Quality of Life 5 Dimensions 5 Levels, HDL, high-density lipoprotein, FSH, follicle stimulating hormone, GGZ Centraal, Psychiatric Center Geestelijke Gezondheidszorg Centraal, GGzE, Geestelijke Gezondheidszorg Eindhoven, ICH-GCP, the International Conference on Harmonization – Good Clinical Practice, IMCJE, International Committee of Medical Journal Editors, iMTA-MCQ, institute for Medical Technology Assessment's Medical Consumption Questionnaire, iMTA-PCQ, institute for Medical Technology Assessment's Productivity Cost Questionnaire, LDL, low-density lipoprotein, LHT, lithium heparin tube, MINI, Mini International Neuropsychiatric Interview Plus, ANSS, positive and negative syndrome scale, PSP, Personal and Social Performance, RvA, Reinier van Arkel Institute for Mental Health Care, TALD, Thought And Language Disorder scale, psychotic disorder NOS, psychotic disorder not otherwise specified, QALYs, Quality Adjusted Life Years, SAE, Serious Adverse Event, SCT, sodium citrate tube, SERM, selective estrogen receptor modulator, SHBG, sex hormone-binding globulin, SMD, standard mean difference, SST, serum separator tube, SUSAR, Suspected Unexpected Serious Adverse Reaction, ULN, upper limit of normal, UMCG, University Medical Center Groningen, UMCU, University Medical Center Utrecht, WOCBP, Women of child bearing potential, ZNA, Ziekenhuis Netwerk Antwerpen, β-HCG, beta-human chorionic gonadotropin

## Abstract

Although acute psychotic symptoms are often reduced by antipsychotic treatment, many patients with schizophrenia are impaired in daily functioning due to the persistence of negative and cognitive symptoms. Raloxifene, a Selective Estrogen Receptor Modulator (SERM) has been shown to be an effective adjunctive treatment in schizophrenia. Yet, there is a paucity in evidence for raloxifene efficacy in men and premenopausal women. We report the design of a study that aims to replicate earlier findings concerning the efficacy of raloxifene augmentation in reducing persisting symptoms and cognitive impairment in postmenopausal women, and to extend these findings to a male and peri/premenopausal population of patients with schizophrenia. The study is a multisite, placebo-controlled, double-blind, randomised clinical trial in approximately 110 adult men and women with schizophrenia. Participants are randomised 1:1 to adjunctive raloxifene 120 mg or placebo daily during 12 weeks. The treatment phase includes measurements at three time points (week 0, 6 and 12), followed by a follow-up period of two years. The primary outcome measure is change in symptom severity, as measured with the Positive and Negative Syndrome Scale (PANSS), and cognition, as measured with the Brief Assessment of Cognition in Schizophrenia (BACS). Secondary outcome measures include social functioning and quality of life. Genetic, hormonal and inflammatory biomarkers are measured to assess potential associations with treatment effects. If it becomes apparent that raloxifene reduces psychotic symptoms and/or improves cognition, social functioning and/or quality of life as compared to placebo, implementation of raloxifene in clinical psychiatric practice can be considered.

## 1 Introduction

Schizophrenia is a common disorder, or rather a collection of several disorders, with heterogeneous combinations of symptoms and variable outcomes [1,2]. While remission of acute psychotic symptoms is achievable for the majority of patients with the use of current treatment options [3], a substantial group of patients remain symptomatic [[4], [5], [6]]. For example negative and cognitive symptoms, which are strongly related to functional outcome, can often persist [5,7]. It therefore remains important to improve pharmacotherapy, for example with augmentation strategies [6,8]. This report describes a study protocol for a novel augmentation strategy to reduce persistent positive, negative and cognitive symptoms and cognitive impairment in women and men with schizophrenia.

In schizophrenia, robust sex differences exist with an incidence risk ratio of 1.4 for men versus women [9,10]. Age of onset is significantly lower in men [2,11], while women show a second incidence peak around menopausal age [12,13]. Premenopausal women experience a more favourable course than men, with less psychotic and negative symptoms, better cognitive and social functioning and less hospitalisations [[14], [15], [16]] [[14], [15], [16]] [[14], [15], [16]]. Women experience less symptoms in the high estrogen phase of their menstrual cycle and higher estrogen levels are strongly correlated with better cognitive performance and lower symptom severity in women with schizophrenia [[17], [18], [19]] [[17], [18], [19]] [[17], [18], [19]]. In men and women with schizophrenia, the estrogen receptor is altered and its expression is lower than in healthy controls [20,21]. Therefore, the ameliorating and protective role of estrogen provides an important lead for new treatment strategies for schizophrenia [14,15,19,22,23].

Although the beneficial effect of estrogen augmentation in the treatment of symptom severity and cognitive impairment in schizophrenia is confirmed by clinical evidence [24], estrogen is not suitable for long term use due to its hazardous side effects on reproductive organs in both sexes and feminizing effects in men. Unlike estrogens, the Selective Estrogen Receptor Modulator (SERM) raloxifene has an estrogenic action in the brain, yet an antiestrogenic action in other tissues. Raloxifene could therefore have therapeutic benefits in patients with schizophrenia of both sexes.

A total of ten trials have been conducted that investigated raloxifene augmentation in schizophrenia, including predominantly postmenopausal women [[25], [26], [27], [28], [29], [30], [31], [32], [33], [34], [35]] [[25], [26], [27], [28], [29], [30], [31], [32], [33], [34], [35]] [[25], [26], [27], [28], [29], [30], [31], [32], [33], [34], [35]]. A recent meta-analysis revealed medium to large effect sizes (ES = 0.32–0.57) of raloxifene compared to placebo on symptom severity [36]. Several studies also suggest that raloxifene augmentation might have beneficial effects on domains of cognitive function [26,34,37,38]. To date, two trials were performed in men and pre/perimenopausal women and/or men [29,30]. Although these trials reported positive effects on symptoms [29] and cognition [30], the beneficial effects of raloxifene cannot be extrapolated to premenopausal women nor to men with schizophrenia without further testing. Such confirmation is essential before implementation of raloxifene in clinical practice can be advised.

In the current report, we describe the study protocol of a double-blind, randomised, placebo-controlled clinical trial examining the effects of 12 weeks of raloxifene augmentation (120 mg/day) on symptom reduction and cognitive improvement in men and pre-, peri- and postmenopausal women with a schizophrenia spectrum disorder. Other outcome measures include general and social functioning. Raloxifene augmentation is expected to reduce positive, negative and general symptoms and to improve cognitive, general and social functioning.

## 2 Methods

This paper is written in line with the Recommendations for the Conduct, Reporting, Editing and Publication of Scholarly Work in Medical Journals (ICMJE) 2014 of the International Committee of Medical Journal Editors (IMCJE) [39]**.**

### 2.1 Aims and objectives

The primary aim of this study is to investigate whether raloxifene augmentation compared to placebo is effective in reducing positive, negative and general symptoms and in improving cognitive functioning. Symptoms are measured by the positive and negative syndrome scale (PANSS) [40] and cognitive function is measured with the Brief Assessment of Cognition in Schizophrenia (BACS) [41]. The BACS consists of 6 tasks measuring different domains of cognitive functioning: list learning (verbal memory), verbal fluency (categorical instances and letter fluency, verbal fluency/processing speed), digit sequencing (working memory), symbol coding (processing speed), Token Motor Task (motor speed/processing speed), and the Tower of London Test (reasoning and problem solving).

Secondary outcome variables include general functioning, measured with the personal and social performance (PSP) [42]; severity of thought disorder, measured with the Thought And Language Disorder scale (TALD) [43]; health-related quality of life and quality adjusted life years (QALYs), measured with the EQ-5D-5L [31]; use of healthcare and non-healthcare resources, measured with the institute for Medical Technology Assessment's Medical Consumption Questionnaire (iMTA-MCQ) and Productivity Cost Questionnaire (iMTA-PCQ) respectively and depressive symptoms, measured with Beck's Depression Inventory (BDI) [32]. The Brief Negative Symptom Scale (BNSS) [44] is used to measure negative symptoms with a higher sensitivity [44].

Genetic, hormonal, and inflammatory biomarkers are assessed to predict treatment response. DNA-samples are collected in saliva. Serum hormonal biomarkers include for women prolactin, follicle-stimulating hormone (FSH) and 17β-estradiol, and for men prolactin, 17β-estradiol, free-testosterone and sex hormone-binding globulin (SHBG). Inflammation is measured as C-reactive protein (CRP) in serum.

Safety data are evaluated by comparing incidences of key Serious Adverse Events (SAEs) and Suspected Unexpected Serious Adverse Reactions (SUSARs) between groups, e.g. hospitalisations.

### 2.2 Trial design and setting

Using a randomised double-blind placebo-controlled multicentre design, we assess the effect of raloxifene augmentation in patients with schizophrenia. After assessing in- and exclusion criteria (see section 2.4.1, 2.4.2), participants are randomised to either raloxifene or placebo treatment. The study is divided in two phases: (1) a treatment phase of 12 weeks and (2) a follow-up phase 2 years. The treatment phase consists of a baseline visit, a midterm visit (6 weeks post-baseline), and an end-of-treatment visit (12 weeks post-baseline). The follow-up phase consists of one visit (6 months post end-of-treatment visit), and two calls (at 12 months and 24 months post end-of-treatment visit). Study examinations scheduled in the course of the study are listed in Table 1. All assessments are performed by the study team, while the treating psychiatrist remains in charge of overall medical treatment. Participants continue their regular treatment during the trial, including antipsychotic medication. Any changes in treatment are recorded during each study call/visit.

**Table 1:** Study procedures.

	Treatment phase				Follow-up phase			
	Visit 1	Call 1	Visit 2	Visit 3	Visit 4	Call 2	Call 3	*
	Baseline	Week 2 of treatment	Week 6 of treatment	End of treatment	Follow-up 6 months**	Follow-up 12 months**	Follow-up 24 months**	Early termination
**Week**	**0**	**2**	**6**	**12**	**38**	**64**	**116**	**-**
Informed consent, in-/exclusion criteria, MINI Plus	**x**							
Medical history, physical examination, presence/regularity of menses	**x**							
Use of concomitant medication	**x**	**x**	**x**	**x**	**x**	**x**	**x**	**x**
Daily functioning and hospitalizations						**x**	**x**	
Dispense study medication	**x**		**x**					
Side effects, compliance		**x**	**x**	**x**				**x**
Blood pressure	**x**		**x**	**x**				**x**
BMI calculation	**x**			**x**				
Smoking/drug/alcohol use	**x**		**x**	**x**				**x**
BACS, EQ-5D-5L, iMTA-MCQ/PCQ	**x**			**x**	**x**			**x**
PANSS, BNSS, TALD, BDI and PSP	**x**		**x**	**x**	**x**			**x**
Blood samples	**x**		**x**	**x**				**x**
DNA (saliva) sample	**x**							

* Early termination visit, performed when a patient prematurely discontinues the study. ** Calculated from end of treatment visit.

MINI, Mini International Neuropsychiatric Interview Plus; BMI, Body Mass Index; BACS, Brief Assessment of Cognition in Schizophrenia; EQ-5D-5L, EuroQol-5 dimensions 5 levels; iMTA-MCQ/PCQ, institute of Medical Technology Assessments' - Medical Consumption Questionnaire/Productivity Cost Questionnaire; PANSS, Positive And Negative Syndrome Scale; BNSS, Brief Negative Symptom Scale; TALD, Thought and Language Disorder Scale; BDI, Beck's Depression Inventory; PSP, Personal and Social Performance scale; DNA, Deoxyribonucleic acid.

### 2.3 Ethical and regulatory standards

This study is performed according to the Declaration of Helsinki and the International Conference on Harmonization – Good Clinical Practice (ICH-GCP). Ethical approval was obtained from the research and ethics committee of the University Medical Center Utrecht (UMCU), protocol 16–103/G-M. The trial is registered in the ClinicalTrials.gov database (identifier NCT03043820) and the European Clinical Trials Database (EudraCT number 2015-004483-11). All study participants complete a written informed consent prior to participation and are eligible to receive other standard of care treatment.

### 2.4 Study population and eligibility criteria

A total of 110 men and women with schizophrenia, schizoaffective or schizophreniform disorder, or psychotic disorder not otherwise specified (NOS) will be included. Recruitment takes place in both in- and outpatient facilities throughout the Netherlands (Institute for Mental Health Care Eindhoven (GGzE), Eindhoven; Psychiatric Center Geestelijke Gezondheidszorg Centraal (GGz Centraal), Amersfoort; UMCU, Utrecht; Reinier van Arkel Institute for Mental Health Care (RvA), ‘s-Hertogenbosch), and one clinic in Belgium (Ziekenhuis Netwerk Antwerpen, ZNA, Antwerp). Patients are approached by their treating psychiatrist and informed about the study by the multidisciplinary study team.

#### 2.4.1 Inclusion criteria

1. Age ≥18 years;
2. Capable of understanding the purpose and details of the study in order to provide written informed consent;
3. DSM-IV diagnosis of: 295. x (schizophrenia, schizophreniform disorder, schizoaffective disorder) or 298.9 (psychotic disorder NOS);
4. Stable dose of antipsychotic medication for at least two weeks;
5. Women of childbearing potential (WOCBP, following menarche and until becoming post-menopausal unless permanently sterile after hysterectomy, bilateral salpingectomy and bilateral oophorectomy) who are sexually active must be willing and capable to use a non-estrogenic contraceptive (e.g. intrauterine device, cervical cap, condom or diaphragm) in case of sexual intercourse during the active treatment phase and at least four weeks after end of treatment
6. Female patients with post coital uterine bleeding must have documented normal Papanicolaou-smear in the preceding five years. If no documented Papanicolaou-smear is present, patients must be willing to undergo one;
7. Female patients between the ages of 52 and 75 must have a reported normal mammogram as part of the Dutch or Belgian breast cancer screening programme in the preceding two years. In case a patient has not participated in the regular breast cancer screening, these patients must be willing to undergo a mammogram.

#### 2.4.2 Exclusion criteria

1. Pre-existing cardiovascular disease (not including hypertension);
2. History of thromboembolic events;
3. History of breast cancer;
4. Familial tendency to form blood clots (such as factor V Leiden);
5. Use of vitamin K antagonists;
6. Use of cholestyramine or other anion exchange resins;
7. Hypertriglyceridemia (triglycerides > 3 times the upper limit of normal (ULN));
8. Liver function or enzyme disorders (serum bilirubin, alkaline phosphatase (AP), gamma-glutamyl transpeptidase (γ-GT), aspartate aminotransferase (ASAT) or alanine aminotransferase (ALAT) > 3 times the ULN as measured at baseline);
9. Severe kidney failure (estimated glomerular filtration rate (eGFR) < 30 ml/min);
10. Use of any form of estrogen, or androgen as hormonal therapy, or antiandrogen including tibolone or use of phytoestrogen supplements as powder or tablet in the past three months;
11. For female patients: pregnancy or breast feeding (serum pregnancy test will be performed at all treatment visits for WOCBP).

### 2.5 Study procedures

During the baseline visit, informed consent is signed and in - and exclusion criteria are checked. The Mini International Neuropsychiatric Interview 5.0.0 is administered to confirm the diagnosis [45]. If the patient is eligible for participation, he or she is randomised and study medication is dispensed. Blood is drawn at several time points to monitor kidney and liver function, lipids and hormone levels (for an overview of all laboratory measures, see Table 2). Furthermore, in WOCBP a pregnancy test is performed during each treatment visit. Two trained experienced researchers interview the patients at each visit using the PANSS and BNSS questionnaires. At all visits, a PSP score is determined and the severity of thought disorder and depressive symptoms is monitored using the TALD and BDI, respectively. Furthermore, side effects are checked and study medication count is conducted during each treatment visit. Cognitive testing, an assessment of quality of life and the use of healthcare and non-healthcare resources is performed at baseline, at end-of-treatment visit (12 weeks post-baseline) and at follow-up visit (6 months post end-of-treatment visit).

**Table 2:** Overview of laboratory measures.

	Visit 1	Visit 2	Visit 3	*
	Screening	6 weeks of treatment	End of treatment	Early termination
**Week**	0	6	12	**-**
Kidney function (eGFR, creatinine)	**x**			
Liver enzymes and function (serum bilirubin, γ-GT, AF, ALAT, ASAT)	**x**	x**	x**	x**
Triglycerides, total cholesterol, HDL- and LDL-cholesterol	**x**	x**	**x**	**x**
CRP	**x**		**x**	**x**
Hormonal biomarkers	**x**		**x**	**x**
***for men:*** prolactin, 17β-estradiol, free testosterone, SHBG				
***for women:*** prolactin, FSH, β-HCG, 17β-estradiol				
β-HCG***	**x**	**x**	**x**	**x**

* performed if a patient prematurely discontinues the study. ** Performed when levels are above the upper limit of normal at baseline. ***Only for WOCBP. LHT, lithium heparin tube; SCT, sodium citrate tube; SST, serum separator tube; eGFR, estimated glomerular filtration rate; ASAT, aspartate aminotransferase; ALAT, alanine aminotransferase; γ-GT, gamma glutamyl transpeptidase; AF, alkaline phosphatase; HDL, high-density lipoprotein; LDL, low-density lipoprotein; CRP, C-reactive protein; APTT, activated partial thrombin time; FSH, follicle stimulating hormone; SHBG, sex hormone-binding globulin; β-HCG, beta-human chorionic gonadotropin

All researchers are trained to conduct the PANSS by a combination of instructional videos and assessment of a test video. Proper conduct of all other rating scales used for this study is trained by experts. Study members are trained regarding Good Clinical Practice. For each participant, visits are conducted by the same study-members.

#### 2.5.1 Patient withdrawal

In case of drop-out during the treatment phase, an early termination visit similar to the end of treatment visit will be performed to finalise participation (Table 1). If the patient is not willing to complete all measures, priority will be given to the PANSS. Possible reasons to terminate a patient's participation in the trial include:

• The patient withdraws his/her consent;
• The patient is intolerant to the study drug;
• The patient receives coercive treatment (based on judicial ruling);
• The patient develops severe kidney failure (eGFR < 30 ml/min), hypertriglyceridemia (>3 times the ULN) or liver disease (defined as serum bilirubin, AP, γ-GT, ASAT or ALAT > 3 times the ULN);
• The patient becomes pregnant;
• Emergence of one or more contraindications against the study drug;
• The investigator considers a patient's continued participation in the study to be unjustifiable on medical grounds (i.e., because of side effects or unusual risks).

If a patient discontinues treatment due to any of the above reasons, the patient receives treatment as usual in normal practice.

### 2.6 Treatment

#### 2.6.1 Group assignment and masking

Randomisation is performed centrally at the Julius Center for Health Sciences and Primary Care, University Medical Center Utrecht. A web-based application with a minimisation procedure is used. Stratification is performed for gender, age and general functioning. The following people are blinded: participants receiving the treatment, persons administering the treatment, persons assessing the outcomes, persons analysing the data. Trial treatment randomisation codes are not available to the study staff, but are available at the local pharmacy for emergency unblinding.

#### 2.6.2 Medication

Participants are randomised to either raloxifene (Raloxifene Teva, EU/I/10/627/001–003), or placebo treatment. The study medication is prescribed by one of the medical doctors involved in this study and taken orally at a daily fixed dosage of 120 mg raloxifene or placebo. Raloxifene Teva 60 mg tablets contain 60 mg raloxifene hydrochloride (HCl), corresponding with 54 mg raloxifene. Placebo tablets filled with the commonly used excipients lactose monohydrate, microcrystalline cellulose and magnesium stearate. Manufacturing of placebo tablets and packaging of the raloxifene and placebo tablets in equal packaging was performed by ACE Pharmaceuticals BV. Raloxifene and placebo tablets have the same external appearance. The study medication is dispensed in two boxes of 92 tablets, handed out at the baseline visit and at visit 2(6 weeks post-baseline).

#### 2.6.3 Treatment duration

Thus far, the duration of trials using raloxifene, in patients with schizophrenia ranged from 6 to 24 weeks [36]. The majority of these trials included specifically postmenopausal women and showed that a treatment duration of 12 weeks is effective in improving symptoms. However, in men and premenopausal women with schizophrenia, raloxifene has only been tested during a period of 8 and 6 weeks, respectively [29,37]. As an intermediate step in the process towards developing long-term treatments for all groups of patients (i.e. pre- and postmenopausal women and men), we decided to give raloxifene during 12 weeks, to be able to investigate prolonged efficacy and monitor the potential risks and side effects in especially men and premenopausal women. When our study proves that 120 mg/day is safe also for men and premenopausal women with schizophrenia, this opens the door to longer trials. These trials could be open-label when efficacy is replicated in the current (double blind) trial.

### 2.7 Safety

Medical history and current medication use are checked at baseline and treatment visits. Blood is screened at baseline for triglycerides, cholesterols, kidney and liver function. If a moderate elevation of triglycerides or liver enzymes is found (<3 times the ULN), these parameters will be measured at each treatment visit.

Physical examination is performed at baseline (visit 1) and end of treatment (visit 3), and blood pressure is measured at baseline and every treatment visit, as hypertension is a known side effect of raloxifene. Pregnancy tests are performed in all WOCBP, at baseline and every treatment visit. In case a patient reports any side effects at any time-point, thorough physical examination will be conducted at the earliest convenience. Furthermore, if appropriate, blood will be drawn. Study continuation or termination is based on the exclusion criteria 7 to 11 as defined in section 2.4.2.

#### 2.7.1 Adverse events

Adverse events (AEs) are defined as any undesirable experience befalling a subject during the course of the study, whether or not it is related to the investigational product. All AEs that are reported by the subject or are observed by the study team are recorded. Side effects and health issues are checked during all treatment visits. The patient will be asked an open-ended question for experience of any side effect or health problems that occurred since the previous visit. The patient fills out the adjusted Green Climacteric Scale [46] and the investigator explicitly questions the occurrence of any thromboembolic event, as these include the most common and most serious side effects of raloxifene. Serious Adverse Events (SAEs) are recorded and reported.

#### 2.7.2 Data and safety monitoring board (DSMB)

A Data Safety Monitoring Board (DSMB) has been implemented in this study. The DSMB consists of experts in the field of the study (i.e. a statistician, a psychiatrist and a gynaecologist) who are completely independent from the study. Members should not have any competing interests that could impact the trial. The DSMB aims to safeguard the interests of trial participants, assess the safety and efficacy of the interventions during the trial, and monitor the overall conduct of the trial. In addition, it aims to assist and advise the sponsor and Principal Investigators in order to protect the validity and credibility of the study, without violating the concepts behind the original protocol. The DSMB receives and reviews the progress and accruing data of the trial on an annual basis. Based on this information, the DSM makes recommendations to the sponsor of the study. Potential recommendations are: no action needed (1), premature termination of the trial (2) or proposing protocol changes (3). Should the sponsor decide not to fully implement the advice of the DSMB, the sponsor will send the advice to the reviewing medical ethical review board; including a note to substantiate why (part of) the advice of the DSMB will not be followed.

### 2.8 Statistical analyses

Unless otherwise specified, a two-sided 0.05 level of significance will be used to declare treatment arms significantly different. Intention-To-Treat analyses will be conducted. All statistical analyses will be performed using SPSS for Windows (version 25.0.0.2) or other widely accepted statistical or graphical software.

#### 2.8.1 Power and sample size calculation

For the sample size calculation, a medium effect size of 0.57 was assumed, which is similar to the effect size for symptom severity detected in the most recent meta-analysis and the effect size for cognition [36,37]. Sample size calculation was based on disjunctive power (or minimal power), which is the probability of finding at least one true intervention effect across all of the outcomes. To be able to find an effect size of 0.57, with two sided alpha set at 5% and with 80% power, 50 participants in each group have to be evaluated. Since we expect some 10% dropout, a total of 55 participants per arm are needed, resulting in a total of 110 participants.

#### 2.8.2 Primary study parameters

The primary analysis will include change in psychotic symptoms as measured by the PANSS at 6 and 12 weeks of treatment (end-of-treatment) and at 6 months after end of treatment and change in cognitive functioning as measured by the BACS at 12 weeks of treatment (end-of-treatment) and at 6 months after end of treatment. A mixed model for repeated measurements will be used, including at least time point, treatment group, the interaction between time point and treatment, sex and age as fixed factors, baseline PANSS/BACS-score, concomitant antipsychotic medication which will be converted to mean daily olanzapine equivalent dose [47], current drug and alcohol use as covariate and subject as random intercept factor. More specifically, the primary analysis will be a repeated measures analysis of variance (ANOVA) with time as within factor (baseline, 6 weeks and 12 weeks of treatment) and treatment as between factor (raloxifene or placebo), with symptom severity (PANSS total, positive, negative or general scores) as dependent variables. For the analysis of cognitive function, the BACS-scores of each of the six domains will be converted into six separate and a composite Z-score for each participant, controlling for age and gender [48]. Separate repeated measures ANOVAs will be performed for the separate *Z*-scores as dependent variables, time as within factor (baseline and 12 weeks of treatment) and treatment as between factor (raloxifene or placebo). The contrast between raloxifene and placebo at 6 and 12 weeks of treatment will be presented with a 95% confidence interval for the difference between the treatment arms. In addition, post-hoc analyses will be conducted to investigate differences at individual time points. There will be corrected for multiple testing by adjusting the p-values produced by each statistical test for each outcome using the Holm method [49,50].

#### 2.8.3 Secondary study parameters

The analyses on other continuous measures will be similar, but note that the number of time points may differ (see also Table 1). Analyses include repeated measures ANOVA with time as within factor and treatment (raloxifene or placebo) as between factor, with the BNSS, PSP, TALD and EQ-5D-5L and use of healthcare recourses as dependent variables. A cost-utility analysis will be performed by dividing costs (both health and non-health) by the QALYs (measured with the EQ-5D-5L). Since costs usually show a skewed distribution, non-parametric bootstrap analyses will provide additional information on the average costs. Differences in total costs between the raloxifene and placebo groups will be related to differences in QALYs.

#### 2.8.4 Additional study parameters

Sex differences and the role of menopausal status in raloxifene efficacy will be explored with regard to the change in positive, negative, general and total symptoms (PANSS) between baseline, 6 weeks and 12 weeks of treatment and change in cognitive function (BACS) between baseline and 12 weeks. These analyses will be performed specifically in the group of patients that received raloxifene treatment, by means of repeated measures ANOVA with time as within factor gender and/or menopausal status as between factor. Furthermore, the analyses on other study parameters, including data from the follow-up visit (6 months after treatment), will be similar to the primary and secondary analyses. With regard to safety data, incidences of key SAEs and AEs will be presented per group. This includes the number and percentage of subjects with at least one occurrence of a SAE or AE. For exploratory purposes, confidence intervals comparing both groups will be provided.

## 3 Discussion

The attainment of full remission of symptoms is one of the major challenges in schizophrenia [6]. Despite current treatment options, diverse positive negative and cognitive symptoms of schizophrenia seem to be permanent and show no improvement over time [[4], [5], [6]]. This study investigates the potential efficacy of raloxifene in order to reduce these persistent symptoms and to improve treatment options for both men and women with schizophrenia. A previous meta-analysis by our group has shown raloxifene to be effective in reducing overall symptomatology [36], yet these findings are based on trials that included predominantly postmenopausal women. Furthermore, research suggests that raloxifene augmentation may be effective in improving cognition in postmenopausal women [26,27,31,33,51], and two trials have shown that this beneficial effect on cognitive functioning may also extend to men and premenopausal women [27,30]. However, the effects on cognition remain inconclusive till date [[33], [34], [35], [36]].

Therefore, we aim to elucidate whether the beneficial effects of raloxifene on positive, negative and cognitive symptoms can be replicated in postmenopausal women, and extrapolated to men and pre-and perimenopausal women with a schizophrenia spectrum disorder. We expect that raloxifene will lower symptom severity and improve cognition when compared to placebo. Furthermore, we expect to find increases in general and social functioning measures since these are highly related to negative symptoms and cognitive functioning [5], and a reduction of use of health and non-health resources.

In 2014, we performed a quantitative review and meta-analysis investigating hormone augmentation strategies in schizophrenia [24]. PubMed, Embase, PsycINFO and Cochrane Library databases were searched for augmentation with estrogens, selective estrogen receptor modulators (SERMs), testosterone, dehydroepiandrosterone (DHEA), pregnenolone and oxytocin in schizophrenia spectrum disorder. The search cut-off date was the last day of November 2014. Only publications on double-blind randomised placebo-controlled trials (RCTs) were included in our analyses. This search revealed that both estrogens and SERMs can be effective strategies for symptom reduction in postmenopausal women with schizophrenia. The SERM raloxifene was shown to have the best side effect profile. Furthermore, raloxifene is currently the only SERM approved for long-term treatment. Kulkarni performed an effective therapeutic dose study [25] for raloxifene augmentation, revealing that a dosage of 120 mg/day was more effective in improving symptomatology than the standard treatment dose of 60 mg/day [25].

In 2018, we performed a quantitative review and meta-analysis investigating raloxifene augmentation in schizophrenia [36], with a search cut-off date of October 2017. A systematic search was performed using PubMed (Medline), Embase, PsychInfo, and Cochrane Database of Systematic Reviews. RCTs investigating the effect of raloxifene in schizophrenia spectrum disorders were included in the quantitative analyses. Outcome measures were psychotic symptom severity, depression and cognition. Nine studies were included, investigating 561 patients with a schizophrenia spectrum disorder. Premenopausal women were included in one study [30], and men were included in two of these studies [29,30]. Raloxifene was superior to placebo in improving total symptom severity (ES = 0.57), as well as positive (ES = 0.32), negative (ES = 0.40), and general symptoms (ES = 0.46). No significant effects of raloxifene were found on cognitive function, although only a small number of studies could be included in these analyses (ranging from 2 to 4 studies for each cognitive domain). These studies included patients that were more severely ill [26,34,35,37], while it has been suggested that greater symptom severity might interfere with improvements in cognitive functioning [52]. Furthermore, a recent study in women with schizophrenia revealed that raloxifene treatment altered performance on specific cognitive domains, dependent on menopause status [27].

### 3.1 Added value of this study

Based on the current literature, there is substantial evidence for the efficacy of raloxifene to reduce symptoms in postmenopausal women with schizophrenia. There is however a paucity in studies of raloxifene efficacy in men and premenopausal women. More evidence is needed to extrapolate its effectiveness and safety profile from this particular patient population towards the entire population of patients with schizophrenia. We therefore aim to replicate recent positive results by conducting a trial in which both men and women with schizophrenia receive raloxifene during 12 weeks in addition to their antipsychotic medication. If we are able to replicate previous positive findings, this evidence may facilitate implementation of raloxifene into general treatment for patients with schizophrenia.

## Funding

This work is funded by ZonMW in the Netherlands (grant number 80-83600-98-40120), as part of the research program Rational Pharmacotherapy (Goed Gebruik Geneesmiddelen), project number 836041008. The funders have no role in the study design, collection, management, analysis and interpretation of data, writing the report, or the decision to submit the report for publication.

## Trial status and registration

The study is currently active and recruiting patients. We anticipate completing of recruitment by September 2020, final assessments (including follow-up visit) are anticipated September 2021. Trial registration: ClinicalTrials.gov with the identifier 2015-004483-11.

## Declaration of competing interest

We declare no competing interests.
